# The Physical Activity Messaging Framework (PAMF) and Checklist (PAMC): International consensus statement and user guide

**DOI:** 10.1186/s12966-021-01230-8

**Published:** 2021-12-19

**Authors:** Chloë Williamson, Graham Baker, Jennifer R. Tomasone, Adrian Bauman, Nanette Mutrie, Ailsa Niven, Justin Richards, Adewale Oyeyemi, Beelin Baxter, Benjamin Rigby, Benny Cullen, Brendan Paddy, Brett Smith, Charlie Foster, Clare Drummy, Corneel Vandelanotte, Emily Oliver, Fatwa Sari Tetra Dewi, Fran McEwen, Frances Bain, Guy Faulkner, Hamish McEwen, Hayley Mills, Jack Brazier, James Nobles, Jennifer Hall, Kaleigh Maclaren, Karen Milton, Kate Olscamp, Lisseth Villalobos Campos, Louise Bursle, Marie Murphy, Nick Cavill, Nora J. Johnston, Paul McCrorie, Rakhmat Ari Wibowo, Rebecca Bassett-Gunter, Rebecca Jones, Sarah Ruane, Trevor Shilton, Paul Kelly

**Affiliations:** 1 Physical Activity for Health Research Centre (PAHRC), Institute for Sport, Physical Education and Health Sciences, University of Edinburgh, Edinburgh, UK; 2 School of Kinesiology and Health Studies, Queens University, Kingston, Canada; 3 Sydney School of Public Health, University of Sydney, Sydney, Australia; 4 Faculty of Health, Victoria University Wellington, Wellington, New Zealand; 5 Sport New Zealand Ihi Aotearoa, Wellington, New Zealand; 6 Department of Physiotherapy, University of Maiduguri, Maiduguri, Nigeria; 7 Department of Health and Social Care, UK Government, London, UK; 8 MRC/CSO Social & Public Health Sciences Unit, University of Glasgow, Glasgow, UK; 9 Sport Ireland, Dublin, Ireland; 10 Ramblers Scotland, Edinburgh, UK; 11 Department of Sport and Exercise Sciences, Durham University, Durham, UK; 12 Centre for Exercise, Nutrition and Health Sciences, University of Bristol, Bristol, UK; 13 Southern Health & Social Care Trust, Portadown, UK; 14 Central Queensland University, Rockhampton, Australia; 15 Department of Health Behavior, Environment and Social Medicine; Faculty of Medicine, Public Health and Nursing, Universitas Gadjah Mada, Yogyakarta, Indonesia; 16 Paths for All, Stirling, UK; 17 University of British Columbia, Vancouver, Canada; 18 Canterbury Christ Church University, Canterbury, UK; 19 Bradford Institute for Health Research, Bradford Teaching Hospitals NHS Foundation Trust, Bradford, UK; 20 Independent Communication Specialist, Waterloo, Canada; 21 Norwich Medical School, University of East Anglia, Norwich, UK; 22 U.S. Department of Health and Human Services, Office of Disease Prevention and Health Promotion, Rockville, USA; 23 Caja Costarricense Del Seguro Social, San José, Costa Rica; 24 Ulster University Doctoral College, Belfast, UK; 25 Centre for Active Living, University of Alberta, Edmonton, Canada; 26 Faculty of Health, York University, York, UK; 27 ParticipACTION, Toronto, ON M5S 1M2 Canada; 28 Sport England, London, UK; 29 National Heart Foundation of Australia, Curtin University, Perth, Australia

**Keywords:** Exercise, Campaigns, Communication, Guidance, Principles

## Abstract

Effective physical activity messaging plays an important role in the pathway towards changing physical activity behaviour at a population level. The Physical Activity Messaging Framework (PAMF) and Checklist (PAMC) are outputs from a recent modified Delphi study. This sought consensus from an international expert panel on how to aid the creation and evaluation of physical activity messages. In this paper, we (1) present an overview of the various concepts within the PAMF and PAMC, (2) discuss in detail how the PAMF and PAMC can be used to create physical activity messages, plan evaluation of messages, and aid understanding and categorisation of existing messages, and (3) highlight areas for future development and research. If adopted, we propose that the PAMF and PAMC could improve physical activity messaging practice by encouraging evidence-based and target population-focused messages with clearly stated aims and consideration of potential working pathways. They could also enhance the physical activity messaging research base by harmonising key messaging terminologies, improving quality of reporting, and aiding collation and synthesis of the evidence.

**Supplementary Information:**

The online version contains supplementary material available at 10.1186/s12966-021-01230-8.

## Introduction

Physical inactivity is a leading cause of non-communicable disease and premature mortality worldwide [1–3]. A systems approach to targeting population level physical inactivity acknowledges that, alongside changes to the physical environment and policy, we must also target social and individual factors such as social norms, perceptions and attitudes [4]. The importance of such approaches are reflected in the Global Action Plan on Physical Activity (2018-2030) [5] and the International Society for Physical Activity and Health’s (ISPAH) eight best investments that work for physical activity (PA) [6]. One example of an approach that can target individual and social factors is PA messaging. We have previously defined PA messaging as “the overall process of creating and delivering PA messages”, with a PA message referring to “educational or persuasive materials to be relayed to a specific individual or group with the aim of ultimately increasing PA levels” [7]. PA messaging is an area of rapidly growing interest [7]. Reflecting this, the World Health Organization (WHO) 2020 guidelines on PA and sedentary behavior include an accompanying paper highlighting the importance of developing effective messaging of guidelines for the first time [8]. Therefore, improving practice in this area is of interest to a range of specialisms including public health, behavioural science, and policy implementation.

To advance PA messaging research and practice, we have developed the PA Messaging Framework (PAMF) and Checklist (PAMC) [9]. Provisional versions of the PAMF and PAMC were developed between March 2019 and April 2020 using concepts identified in a scoping review of PA messaging [7], drawing from relevant theory and existing frameworks [10, 11] and through consultation with researchers, policymakers and practitioners. The provisional framework and checklist provided a starting point in a modified Delphi study [9]. In this Delphi study, we conducted three mixed methods online surveys to gather feedback from an international expert panel (*n* = 40, 55% female) comprising academics (55.0%), healthcare professionals or other professionals (22.5%) and government officials or policymakers (22.5%). The framework and checklist were amended and developed between each survey round based on feedback until consensus (defined a priori as 80% agreement) was reached from the panel [9].

A detailed report of the modified Delphi methods and results have been published in a separate paper [9]. The current paper presents the resulting consensus statement with accompanying user guide for the PAMF and PAMC. This approach was taken to maximise usefulness and facilitate implementation, and is consistent with Guidance on Conducting and Reporting Delphi Studies (CREDES) [12]. This consensus statement and user guide may enable researchers, practitioners, and others to adopt and use the PAMF and PAMC consistently. If adopted by the PA for health field and used consistently, the PAMF and PAMC have potential to improve PA messaging practice and strengthen the PA messaging research base.

### Aims

In this consensus statement and user guide, we aim to: (1) present an overview of the various concepts within the PAMF and PAMC; (2) discuss how the PAMF and PAMC can be used to create PA messages, plan evaluation of messages, and aid understanding and categorisation of existing messages; and (3) describe areas for future development and research.

### The physical activity messaging framework (PAMF) and checklist (PAMC)

#### Overview of the framework and checklist

**Fig. 1:**
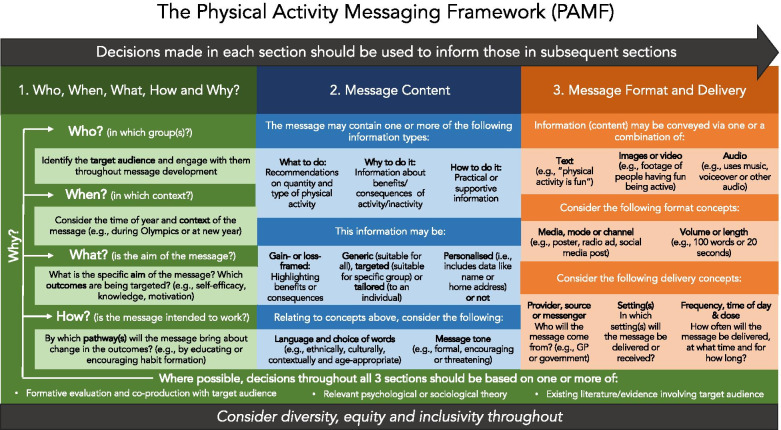
The Physical Activity Messaging Framework (PAMF)

Figure 1 and Additional File 1 present the PAMF and the PAMC respectively. The PAMF presents an overview of messaging concepts for each overarching section and provides a visual tool for communications, teaching, and training. The PAMC presents these concepts in a more practical format and acts as a tool for implementing the framework that can be used to guide and document message creation, evaluation, and categorisation. Working definitions of concepts within the PAMF and PAMC can be found in Table 1.

**Table 1:** Working Definitions of Key Concepts within the PAMF and PAMC

Concept	Working definition	Example(s)
**Concepts relating to Section 1: Who, when, what, how and why?**		
Target audience	The intended recipient(s) of the message	Older adults, individuals working from home
Context [13]	The time of year and the geographical, epidemiological, socio-cultural, socio-economic, ethical, legal and political context at the time of messaging	During the winter; at New Year; during a global pandemic
Outcomes [14]	Changes expected as a result of messaging	Awareness, understanding, motivation, physical activity behaviour
Pathway [14]	The sequential process from the delivery of the message through to outcome. In other words, *how* a message works. This may encompass multiple mechanisms or processes.	Education, persuasion, encouraging habit formation, targeting beliefs about capabilities
Formative research/evaluation [15]	Evaluation or research used to help inform message development and to assess whether the message is needed, appropriate and acceptable before it is implemented.	Focus groups or interviews with members of the target population to investigate message salience, relevant and importance
Co-production (Smith B, Williams O, Bone L: Co-producing research in the sport, physical activity and exercise sciences: A resource to guide co-production, forthcoming).	Bringing together citizens with those working in research, policy and industry, and/or practice in an attempt to form equitable partnerships throughout message development	Involving individuals from the target audience in message development
**Concepts relating to Section 2: Message content**		
‘What to do’ information	Information regarding the amount or type of physical activity that is recommended	150 min of moderate physical activity per week; 10,000 steps per day; a mixture of aerobic and strength activity
‘Why to do it’ information	Information regarding benefits (or consequences) of physical activity (or inactivity)	Physical health, mental health, appearance, environment
‘How to do it’ information	Information providing guidance on how to be more active or signposting to opportunities for physical activity.	Guidance on when to be active, where to be active or who to be active with
Use of gain- or loss- framing [16]	The use of framing a message to highlight either the benefits of taking part in physical activity (gain-framed) or the consequences of not taking part (loss-framed).	Gain-framed: “Walking regularly can make you happier” Loss-framed: “Not walking regularly can increase your risk of depression”
Tailoring [17]	Information based on individual data	Specific feedback on pre-established goals such as step counts
Targeting [17]	Information designed to be relevant to a specific group	Information relevant to inactive individuals or people with Diabetes
Personalisation [17]	The use of static, individual-specific information in a message	Messages involving name or home address
Language and choice of words	The dialect(s) and selection of specific wording used in the message	English, Spanish, use of slang, use of lay-audience friendly language
Message tone	The tone adopted by the message	Threatening, persuasive, encouraging
**Concepts relating to Section 3: Message format and delivery**		
Text (message format)	The use of words to convey information in a message	Text on posters or social media posts
Images or video (message format)	The use of images and videos to convey information in a message.	Images or footage of individuals being active
Audio (message format)	The use of audio to convey information in a message.	Music, voiceovers, sound effects
Media, mode or channel of delivery	The type of media through which the message is being communicated	Emails, posters, social media posts, radio/television adverts
Message volume or length	The volume or the length of the message relating to the number of words in a message or the amount of time it takes to listen to a message.	100 words, 20 s audio/video clip
Provider or source	The provider or source of the message	Doctor, journalist, reporter, friends/family
Setting	The setting in which the message will be received by the intended recipient	Doctor’s waiting room, home, work, school
Frequency, time of day and duration	How often the message is delivered, at what time, and for how long. Together these contribute to the overall dose of message delivered.	Emails sent in the morning 3 times a week for 4 weeks

Table adapted (with permission) from Williamson et al. [9]. Where cited, definitions adapted from source to align specifically with PA messaging

#### Section 1: Who, when, what, how and why?

Throughout this paper, we use the term ‘user’ to describe the individual(s) using the PAMF and PAMC to create, evaluate or understand PA messages. The *Why?* concept extends along the length of the framework and, although placed within section 1 for clarity, is relevant for all concepts within the framework. This section asks the user to first consider, explain and justify “why” section 1 decisions, and then in a sequential manner “why” subsequent section 2 and 3 decisions on content, format, and delivery, were made. Drawing on theory to develop and understand health messaging is likely to improve planning and targeting, help define more explicit message aims and potential pathways, and ultimately result in more effective messages [18]. Existing evidence supports conducting formative evaluation (see Table 1) with the target audience and drawing on psychological theory and social marketing principles in the message development [7]. However, as with many other health promotion programmes [18], message creators often design and implement the message without conducting formative research or sufficiently understanding the target population [7]. Furthermore, many PA messages are created without establishing a clear aim and without drawing on theory to inform message development [7]. *Why?* encourages the user to have a clear rationale for each decision by making choices based on formative evaluation and co-production with the target audience, relevant psychological or sociological theory, and/or existing evidence involving the target population.

*Who?* encourages the user to identify and specify a target audience at the outset of message development and to continue engaging with them at all stages of message creation and delivery. For example, is the message aimed at older adults, inactive populations, those in the ‘pre-contemplation’ stage of change, or children in a specific region? A recent paper on maximising impact of PA guidelines through communication approaches presented a planning framework which shows the importance of situational and stakeholder analyses to identify appropriate target groups [8]. Dividing the general public into subgroups with similar characteristics/variables or ‘audience segmentation’ is a key element of social marketing and an important early step in developing targeted health communications [19]. There are numerous ways in which a population can be ‘segmented’, for example, by sociodemographic, geographical, behavioural, epidemiological, attitudinal or psychological variables [18], by peer crowds [20], or combinations of these variables. We acknowledge that while segmentation is advised, some messages may be targeted at numerous groups or a general population, for example in a national mass media campaign. Engaging with the target audience(s) through formative evaluation and co-production can provide an understanding of their attitudes, circumstances, challenges and preferences [18]. While such approaches may not always be viable or appropriate [21], they offer an opportunity to develop messages that are relevant and salient to the target group, and thus have a higher chance of success [18].

*What*? encourages the user to identify specific aim(s) of the message(s) and, linked to this, state what the message is trying to achieve in terms of proximal, intermediate and distal outcomes [7]. For example, does the message aim to raise awareness or knowledge of PA benefits in older adults, or improve self-efficacy in teenage girls? Relatedly, *How?* encourages the user to state how these outcomes will be achieved by the chosen message(s), that is by which pathway(s) [10] or process(es)? It may be particularly useful here to refer to existing theory, such as the Transtheoretical Model, Social Cognitive Theory or the Behaviour Change Wheel [10, 11, 22–25], to identify plausible ways in which the message might bring about changes in the outcome(s) of interest. For example, with reference to behaviour change theory [11], targeting ‘beliefs about capabilities’ (mechanism of action) may be used to bring around change in self-efficacy (outcome). Or utilising ‘education’ (intervention function) within a message in the form of providing information on health benefits of PA (behaviour change technique) may bring about a change in knowledge (outcome).

Finally, interrelated with all other concepts in section 1 (*who*, *what*, *how* and *why*), *When?* considers the time of year and context in which the message is created and delivered. For example, some message developers may wish to capitalise on certain times when goals are more likely to be set such as new year, or when fewer barriers to PA are present such as during summer months when weather is better and there are more daylight hours [26, 27]. Context (such as epidemiological, social or political context) [13] may influence what is perceived as important to the target audience and what is feasible to promote. A recent example is the COVID-19 pandemic, during which government guidance has influenced which types of PA can be promoted [28]. During the pandemic, factors most important to the target audience may have shifted from, for example, appearance and physical health to social and mental health. Additionally, many individuals working at home may not have regular access to some delivery channels, such as workplaces and billboards. It is therefore important to consider context when deciding what information should be included in the message and how it should be delivered.

#### Section 2: Message content

Section 2 has three levels. The first level encourages the user to consider the type of information in the message, of which there are three potential types: (1) *what to do*, (2) *why to do it*, and (3) *how to do it*. Examples of these three information types can be found in Table 2. *What to do* information includes information on amount, intensity and type of PA being promoted. For example: information on the PA guidelines such as 150 min of moderate-to-vigorous PA per week [29–32], 30 min of PA on most days of the week [33], or step count recommendations such as 10,000 steps per day [34]. *Why to do it* information includes information on any benefits (or consequences) of being active (or inactive). This information can relate to a number of areas, such as physical health [2, 3], mental health [35, 36], appearance [37, 38] or the environmental impact of PA [39], and may refer to immediate, short term and/or long-term effects [7]. Current evidence points towards the benefits of promoting immediate short-term benefits of PA, particularly relating to affective state and mental or social health [7]. However, formative research and co-production with each target audience may further reveal what information is most salient and important. Finally, *how to do it* information encompasses practical or supportive information that may provide instructions or guidance on how, when, and where to be active, e.g., signposting to local opportunities. Note that a PA message may include just one or a combination of these information types, and does not necessarily need to include information on the PA guidelines [7].

**Table 2:** Examples of different information types in physical activity messages

Information type	Examples
What to do	“Adults should aim to accumulate 150 min of moderate- to vigorous- physical activity a week” “Aim for 10,000 steps a day or more” “Aim to take part in both aerobic and strength exercises”
Why to do it	“Being physically active can reduce your risk of heart disease later in life” “Take the stairs – feel less stressed” “Cycle for a healthier planet” “A little movement for a little mood improvement”
How to do it	“Try walking during your lunch break to become more active!” “Set weekly goals and smash them!” “Did you know that we run a group walk for University staff every Thursday at 12 pm? It starts outside the library. Why not come along next week?”

The second level of section 2 relates to the way the information is conveyed. It considers (1) information *framing*, (2) the use of *generic*, *targeted, or tailored* messages, and (3) the use of *personalisation*. PA message *framing* relates to whether information is framed to highlight the benefits of taking part in PA or the consequences of not taking part [16]. Framing involves both the exposure (PA) and the outcome. For example, where *gain-framed* messages may be: “regular activity can improve your heart health” or “walking daily is good for your mental health”, *loss-framed* alternatives would be: “inactivity increases your risk of dying of heart disease” or “not walking daily may increase your risk of depression”. Existing evidence generally supports the use of gain-framed messages over loss-framed messages to promote PA [7, 16], however, engaging with the target audience may highlight instances where there is no benefit of framing [40] or where loss-framed messages are preferred. For example, there is evidence to suggest that people with spinal cord injury can be motivated to engage in PA by increasing risk perception through loss-framed messaging [41, 42].

**Fig. 2:**
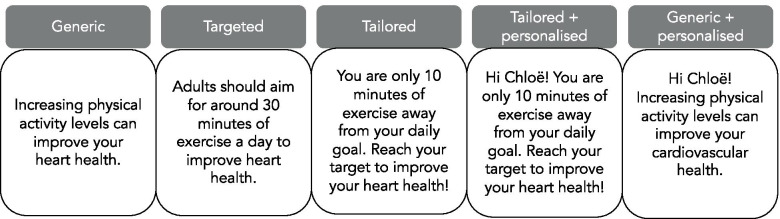
Illustrative examples of combinations of tailored, targeted, and personalised messages

Information in a PA message may be *generic*, *targeted* (at a group level) or *tailored* (at an individual level). *Generic* information is intended to be suitable for all audiences and may include, for example, information on generic benefits of PA or PA opportunities [7]. *Targeted* messages are relevant to a particular group [17]. For example, a targeted message aimed at older adults may specifically highlight benefits of PA which are particularly relevant to that group, such as spending time with others and maintaining functional capacity [43]. *Tailored* messages include user-specific data [17] such as goals to make messages highly relevant for that individual. For example, messages conveying how close someone is to meeting their personal step count goal. Generally, existing evidence supports the use of targeted or tailored messages over generic messages [7]. Finally, *personalising* a message includes using non-PA related data [17] such as name or home address to increase salience of the message. Figure 2 shows how *targeting*, *tailoring* and *personalisation* can be used alone or in various combinations.

The final level of section 2 relates to the *language* used in the message. The user is encouraged to consider if the language used is ethnically, culturally, regionally, literacy and age-group appropriate for the target population. It is important that message content demonstrates an understanding of cultural sensitivities [44], and message credibility and appeal may be increased when messages reflect the social and cultural world of the target audience [19]. The user is also encouraged to consider message *tone.* For example, is a formal or encouraging tone suitable for the target population and message aim(s)? Can threatening, condescending, or demanding tones be recognised and avoided? Existing evidence suggests threatening or forceful tones are at best ineffective and at worst may have detrimental effects on PA and PA-related outcomes such as intentions, motivation and affect [45–47].

#### Section 3: Message format and delivery

The final section of the PAMF and PAMC relates to message format and delivery. First, the user is encouraged to consider how the content of the message may be conveyed: via *text* or *words*, for example “physical activity is fun!”, using *images* or *video*, for example showing footage of people having fun being active, or using *audio*, for example including a voiceover or using ‘fun’ music in the message. Second, the user is encouraged to consider message format by considering both the *media, mode* or *channel* of the message (e.g., poster, Instagram post, radio advert), and the message *volume* or *length* (e.g., 100 words or 20 s). Existing evidence suggests message format preferences vary from group to group [7]. It is therefore important to draw on existing literature and utilise formative evaluation to inform such decisions.

Finally, the PAMF and PAMC guide the user through a series of delivery concepts. The first is the message *provider*, *source* or *messenger* (e.g., the Government, healthcare professionals, a certain organisation, or a credible role model or celebrity). Message provider characteristics (such as appearance, gender, age, organisation credibility etc) may impact the acceptability of a message [48, 49], and different populations have different message provider preferences [7]. Some populations may value credible information from experts, whereas others may find expert advice overpowering [19], once again highlighting the importance of formative evaluation and co-production with the target audience. The *setting(s)* should be considered for message delivery, such as doctor’s office, at home or at work. Finally, the framework encourages (where relevant) consideration of *frequency, time of day and duration* of the message. For example, a Tweet that is sent 3 times a week between 9 and 10 am and will be sent for 6 months. The PAMF and PAMC link message delivery and format decisions with message aim(s), target audience, and what is most appropriate based on theory, formative research and/or existing evidence.

#### Ensuring equity, diversity, and inclusivity in the messaging process

Addressing inequalities is a well-known challenge in PA promotion [50–53], and therefore considering diversity, equity and inclusivity when creating PA messages is crucial. It is important to consider equity when creating and delivering PA messages and aim to avoid creating or worsening biases between groups that differ socially, economically, demographically or geographically. We can learn from previous communication efforts in other health behaviours here. For example, smokers from more deprived neighbourhoods with higher smoking prevalence are less equipped to change behaviour in response to anti-smoking promotions [54]. Similarly, consistent with the knowledge gap deficit model [55, 56], evidence from the Canadian ParticipACTION campaign suggests that individuals with a higher level of education have higher motivation to attend to PA health messages [57]. Therefore, not due to individual choice but rather due to social disadvantage, some groups may need more practical advice on how to increase PA, have differential access to social media, or may not have safe green spaces nearby to act on messages they see. Indeed, in some groups, messaging may not be an appropriate or priority strategy to target PA. It is therefore important that we utilise formative evaluation to assess the need for messaging, adapt message content and delivery based on what will work best for each population where messaging is appropriate, and continue to view messaging as part of overall PA promotion.

Although we may aim to target messages to specific groups, these target audiences are not homogeneous. Therefore, to ensure messages reach and appeal to diverse groups it is critical to involve and consider individuals from a range of different sociocultural backgrounds in message creation where appropriate to gather as many viewpoints as possible [58]. Some cultural adaptation models suggest having researchers (in this case, message co-creators) of similar cultural backgrounds to that of the target population [59]. Furthermore, existing evidence suggests that individuals may respond more positively to messages with relatable content and models in their images/videos [7, 44, 60]. Similarly, when using images and video footage in PA messages, it may be important to represent the various individuals in that target audience by including, for example, individuals from various social and cultural backgrounds, different genders, body types, fitness levels and sexual orientations. One example is the This Girl Can campaign [61] which targets the population of ‘women in England’. Developed as a result of formative research with various subgroups of women, the final campaign images and videos used models who represented a broad range of women, enhancing relatability [62, 63]. Alternatively, message creators may wish to avoid using models at all and use more generic icons or images instead. This approach was taken recently in the logo of the Move Your Way® campaign (USA) [64].

Finally, PA messages should cater for marginalised groups in society as well as mainstream audiences [18], using inclusive language and accessible delivery formats. The importance of considering inclusivity in PA messaging has been highlighted by a recent editorial [65] in which the authors explain how some commonly used PA messages aiming to tackle physical inactivity and sedentary behaviour such as ‘sit less, move more’ are ableist. Working with often overlooked or marginalised groups to co-produce messages will ensure inclusivity. Indeed, the PAMF’s emphasis on formative evaluation and co-production can contribute towards addressing this.

### Application of the PAMF and PAMC

#### Using the PAMF and PAMC to create new messages

One use of the PAMF and PAMC is to create new messages. These could include standalone messages or, for example, a group of messages to be included in a mass media campaign. When creating messages, the PAMF and PAMC are intended to be used sequentially with decisions in section 1 being used to help inform subsequent decisions. The checklist can be used throughout the message creation process to ensure all relevant concepts within the framework have been considered and to document this process. There may be different levels at which an individual or organisation uses the framework and checklist. At one end of the scale, the user(s) may have their own established messaging approach and may simply wish to use the PAMF and PAMC in a ‘light-touch’ way to inform or check their process. On the other end, the user(s) may wish to be prescriptively directed by the PAMF and PAMC from start to finish.

Where the PAMF and PAMC are being used in a more prescriptive way, new messages may be created using an interdisciplinary team of academics/researchers and practitioners/professionals and consumers, systematically considering each concept in the framework, and drawing on each group’s strengths to inform various decisions. However, we acknowledge that practically speaking this may not always be possible, and that there will likely be situations where resource realities (restrictions on time, personnel, and funding etc) will limit the level of framework consideration. Indeed, demonstrations of pragmatic use of the PAMF and PAMC in various circumstances with varying levels of resources will provide valuable insight into their implementation in practice-based settings [66]. We believe that messages created with at least some consideration of the framework will be more effective than those that have not considered any of the included concepts.

In some cases, it is plausible that a brief has been issued or some key decisions regarding content and delivery have already been made. For example, a university may task the user with designing a poster to encourage students to use the stairs in the library. Here, some aspects around message aim, format and setting have been decided. The PAMF and PAMC can still be used to record which decisions were pre-specified, and which decisions and concepts were subsequently considered and guided by the framework. Alternatively, you may be given the brief of developing the communication strategy for national PA guidelines. In this case the PAMF and PAMC can be used prospectively to guide a range of options and approaches, identifying which may have the best supporting evidence.

Although the PAMF is designed to specifically aid PA messaging, there may be parallels and overlaps with other sub-types of PA communication, or approaches that rely heavily on communication, such as public lectures, counselling, or advocacy [8]. We encourage the use of principles from the PAMF in other types of PA communication where appropriate and useful.

#### Using the PAMF and PAMC to evaluate messages

The PAMF and PAMC may also assist with the evaluation of PA messages (see Table 3). In formative evaluation, the framework and checklist may aid in planning research with the target audience to help understand the need for messages in that group and inform the development of new messages. As highlighted throughout, concepts from the framework may be used to guide development of data collection methods in qualitative or quantitative research (e.g., focus group topic guides or questionnaires) exploring messaging preferences. The framework and checklist themselves are not tools to conduct process or impact/outcome evaluation of messages but may help identify important indicators of message success and therefore aid in the development and planning of process and impact/outcome evaluation.

**Table 3:** Types of evaluation

Evaluation type	Working definitions (adapted from Bauman & Nutbeam, 2014) [15]
Formative evaluation of physical activity messaging	Gathering data to help inform message development and to assess whether the message is needed, appropriate and acceptable before it is implemented. (e.g., using focus groups to test alternate messages, and establish messaging preferences).
Process evaluation of physical activity messages	Establishing whether or not the message was delivered as intended (e.g., what was the message reach? Was the message delivered successfully to the intended target audience, at the correct time, in the desired setting?)
Impact/Outcome evaluation of physical activity messages	Establishing whether or not the message produced changes in the desired indicators (e.g., did the message bring about changes in awareness, attitudes, or physical activity behaviour?)

#### Using the PAMF and PAMC to understand and classify messages

Using the framework and checklist as classification tools may be useful in a range of scenarios. The framework and checklist may be useful in retrospectively classifying and comparing existing messages to understand the features included. This may assist in identifying concepts that were not considered, highlighting which messaging concepts are most important and providing direction for future research. For example, if two existing mass media campaigns both aimed at the same target audience had varying levels of success in improving perceptions towards PA, we may use the checklist to deconstruct and classify included messages to identify effective components. Similarly, the PAMF and PAMC may be used to classify or compare messages regarding various elements or formats of national or international PA guidelines. For example, messages comparing various formats of the aerobic guidelines (150 min per week, 2.5 h per week or 30 min 5 times per week), or messages highlighting the aerobic guidelines versus those highlighting strength and balance guidelines. Another scenario may be using the checklist to categorise different messages included in a systematic review of PA messaging or in an existing mass media campaign. The use of the PAMF and PAMC as classification tools may also help improve quality of message reporting going forward, ultimately enhancing the messaging evidence base.

#### Potential benefits of framework and checklist

Overall, the PAMF and PAMC aim to harmonise and enhance the area of PA messaging. Specifically, we propose that the framework and checklist may have five potential benefits. First, they provide an illustration of important and common PA messaging concepts that could be considered when creating, evaluating or categorising PA messages. Second, they may standardise and facilitate our understanding and use of key PA messaging terminologies and concepts. Third, they encourage engagement with target audiences and the use of relevant theory and existing evidence in message development. Fourth, they aim to address the often missing step of stating and understanding working pathways in the process of messaging in PA behaviour change and designing and evaluating messages accordingly. Finally, the PAMC provides a translational checklist tool that can be used by academics, practitioners, and any other relevant stakeholders to develop and evaluate PA messages.

### Future directions

For all different uses, the level of engagement with PAMF and PAMC will vary based on available resources. It is highly plausible in applied scenarios (beyond academic settings) that rapid message creation or evaluation is needed. In such situations, perhaps the PAMF and PAMC will only provide “top level” guidance. Exploring how to facilitate this is a key priority moving forward.

Improving functionality and usefulness of the PAMF and PAMC for various groups of users are also key future directions. Developing an online interactive tool may be helpful in improving usefulness of the PAMF and PAMC for different groups of users and make documenting the messaging process more comprehensible. Making training available to facilitate the adoption and uptake of the PAMF and PAMC may also be a useful future direction.

The PAMF and PAMC presented in this article have consensus from a group of international experts, but may evolve further, along with the working definitions of included concepts. Similar to the evolution of existing reporting guidelines (e.g. PRISMA [67] and CONSORT [68]), the PAMF and PAMC will be revised based on their use in applied settings and future examination in academic study. Furthermore, although the PAMF and PAMC were developed with input from a multidisciplinary panel, it may need terminology adaptation in cross-disciplinary settings, for example in media disciplines.

Future research may retrospectively evaluate messages to illuminate important or effective concepts or test the effectiveness of messages created using the PAMF/PAMC (versus those created not using the PAMF or control messages) in different trial designs. Furthermore, global, and national PA guidelines now also include reference to reducing sedentary behaviour. Indeed, recent 24-h movement guidelines for Canadian adults have faced a new challenge of creating messages not only for PA guidelines, but for integrated guidelines that cover sleep, sedentary behaviour and PA [69]. Future research may therefore also explore the applicability of the PAMF in creating and guiding evaluation of messages focusing on related health behaviours such as sedentary behaviour and sleep either combined with PA messages or independently.

## Conclusion

Effective PA messaging plays an important role in the pathway towards changing PA behaviour at a population level. In this article we have described the outputs of a recent modified Delphi study, the Physical Activity Messaging Framework and Checklist, and discuss how they can be used to create new messages, plan message evaluation, and help understand and categorise existing messages. If used consistently, we propose that the framework and checklist have potential to improve PA messaging practice by encouraging evidence-based and target population-focused messages. Further, this framework and checklist could augment PA messaging research by improving quality of reporting, harmonising messaging terminologies and aiding collation and synthesis of evidence.

## Supplementary Information

**Additional file 1.**

## Data Availability

The Physical Activity Messaging Framework is available as a figure in this paper. The Physical Activity Messaging Checklist is available as a downloadable supplementary file.

## Abbreviations

PA: Physical activity
PAMF: Physical Activity Messaging Framework
PAMC: Physical Activity Messaging Checklist
